# Poster Session I - A169 COMPARATIVE OUTCOMES OF EMR AND ESD FOR BARRETT’S NEOPLASIA

**DOI:** 10.1093/jcag/gwaf042.169

**Published:** 2026-02-13

**Authors:** H Abdulhussain, Y Fujiyoshi, A Rostom

**Affiliations:** Gastroenterology and Hepatology, University of Ottawa Faculty of Medicine, Ottawa, ON, Canada; Gastroenterology and Hepatology, University of Ottawa Faculty of Medicine, Ottawa, ON, Canada; Gastroenterology and Hepatology, University of Ottawa Faculty of Medicine, Ottawa, ON, Canada

## Abstract

**Aims:**

EMR and ESD are established endoscopic therapies for Barrett’s neoplasia. ESD provides potential advantages in achieving en-bloc and curative resections but is less commonly performed in North American centers. This study compared histologic, clinical, and safety outcomes between ESD and EMR for Barrett’s neoplasia at a tertiary Canadian center.

**Methods:**

We conducted a retrospective analysis of patients undergoing EMR or ESD for Barrett’s neoplasia between 2017 and 2025 at The Ottawa Hospital.

Primary outcomes were en-bloc, R0, and curative resection rates (defined as R0 resection with high-grade dysplasia or well-to-moderately differentiated intramucosal carcinoma, no lymphovascular invasion, and submucosal invasion ≤500 μm).

Secondary outcomes included persistent or recurrent dysplasia, complete remission of dysplasia (CRD), complete remission of intestinal metaplasia (CRIM), and procedure-related adverse events (AEs). Clinically significant AEs included post-procedural events such as perforation, stricture, or deep mucosal injury requiring endoscopic intervention.

**Results:**

33 patients were included (ESD = 13; EMR = 20), with a median age of 67 years; 84.8% were male. ESD lesions were significantly larger (2.5 [2.0-3.0] cm vs. 1.0 [0.8-1.5] cm, P = 0.04). En-bloc resection was achieved in 100% of ESD versus 55% of EMR (P = 0.005). R0 resection rates were 76.9% (ESD) and 60.0% (EMR; P = 0.46), while curative resection was achieved in 69.2% and 50.0%, respectively (P = 0.31). Persistent dysplasia occurred in 15.4% of ESD and 30.0% of EMR cases (P = 0.29). Among patients followed ≥12 months, CRD was achieved in 84.6% (ESD) and 70.0% (EMR), while CRIM was achieved in 69.2% and 60.0%, respectively. No recurrence of dysplasia was observed after CRD in either group. Adverse events occurred in 15.4% of ESD and 5.0% of EMR patients (P = 0.58).

In the ESD group, one patient developed a post-procedural stricture, and one experienced a deep mucosal injury managed endoscopically.

In the EMR group, one patient developed a post-procedural stricture. There were no perforations, bleeding, or procedure-related deaths. Three patients (9.1%) underwent esophagectomy for non-curative histology rather than recurrent disease (two after ESD, one after EMR).

**Conclusions:**

ESD was preferentially used for larger and more advanced Barrett’s lesions, achieving higher en-bloc, R0, and curative resection rates compared with EMR, with similar safety when clinically meaningful adverse events were considered.

Although ESD was associated with isolated cases of stricture and deep mucosal injury, all were managed endoscopically without morbidity or mortality. These real-world findings support ESD as the preferred approach for larger or advanced Barrett’s neoplasia when en-bloc resection is critical, while EMR remains a safe and effective option for smaller, less advanced lesions.

**Funding Agencies:**

None

